# Prediction of Readmission in Geriatric Patients From Clinical Notes: Retrospective Text Mining Study

**DOI:** 10.2196/26486

**Published:** 2021-10-19

**Authors:** Kim Huat Goh, Le Wang, Adrian Yong Kwang Yeow, Yew Yoong Ding, Lydia Shu Yi Au, Hermione Mei Niang Poh, Ke Li, Joannas Jie Lin Yeow, Gamaliel Yu Heng Tan

**Affiliations:** 1 Nanyang Business School Nanyang Technological University Singapore Singapore; 2 City University of Hong Kong Hong Kong Hong Kong; 3 School of Business Singapore University of Social Sciences Singapore Singapore; 4 Tan Tock Seng Hospital Singapore Singapore; 5 Geriatric Education and Research Institute Singapore Singapore; 6 Ng Teng Fong General Hospital Singapore Singapore; 7 Medical Informatics National University Health System Singapore Singapore

**Keywords:** geriatrics, readmission risk, artificial intelligence, text mining, psychosocial factors

## Abstract

**Background:**

Prior literature suggests that psychosocial factors adversely impact health and health care utilization outcomes. However, psychosocial factors are typically not captured by the structured data in electronic medical records (EMRs) but are rather recorded as free text in different types of clinical notes.

**Objective:**

We here propose a text-mining approach to analyze EMRs to identify older adults with key psychosocial factors that predict adverse health care utilization outcomes, measured by 30-day readmission. The psychological factors were appended to the LACE (Length of stay, Acuity of the admission, Comorbidity of the patient, and Emergency department use) Index for Readmission to improve the prediction of readmission risk.

**Methods:**

We performed a retrospective analysis using EMR notes of 43,216 hospitalization encounters in a hospital from January 1, 2017 to February 28, 2019. The mean age of the cohort was 67.51 years (SD 15.87), the mean length of stay was 5.57 days (SD 10.41), and the mean intensive care unit stay was 5% (SD 22%). We employed text-mining techniques to extract psychosocial topics that are representative of these patients and tested the utility of these topics in predicting 30-day hospital readmission beyond the predictive value of the LACE Index for Readmission.

**Results:**

The added text-mined factors improved the area under the receiver operating characteristic curve of the readmission prediction by 8.46% for geriatric patients, 6.99% for the general hospital population, and 6.64% for frequent admitters. Medical social workers and case managers captured more of the psychosocial text topics than physicians.

**Conclusions:**

The results of this study demonstrate the feasibility of extracting psychosocial factors from EMR clinical notes and the value of these notes in improving readmission risk prediction. Psychosocial profiles of patients can be curated and quantified from text mining clinical notes and these profiles can be successfully applied to artificial intelligence models to improve readmission risk prediction.

## Introduction

### Background

Hospital readmission of older adults is a significant challenge for the individual, caregivers, and health system. For individuals, readmissions can be distressing, may compromise quality of care, and increase the risk of adverse health outcomes. For caregivers, readmission is often burdensome and increases their health care spending. As for health systems, readmissions often cause resource demands and financial costs to escalate [1]. The 30-day readmission rate among patients aged 65 years or older in Singapore has been reported to be 19% [2], which is comparable to the readmission rate of Medicare patients in the United States, most of whom are older adults [3]. Significant risk factors for hospital readmission in adults aged 65 years and older include (a) sociodemographic factors such as higher age, male gender, ethnicity, and poor living conditions; (b) health-related factors such as poor overall condition, comorbidity, functional disability, and recent hospital admissions; and (c) organizational factors such as prolonged length of stay in the index hospitalization and discharge destination [4,5]. These risk factors have been used extensively in predictive models for hospital readmission by health service researchers worldwide [6-10]. Recently, other readmission predictors such as those in the psychosocial domain have begun to receive more attention.

Psychosocial factors can be defined as “the combination and interplay of psychological and social factors that potentially influence health, injury, illness, and disease” [11]. However, a review of the medical literature suggests that different medical specialties have slightly different definitions of psychological factors [11-17]. Based on the various factors identified in earlier studies, we observed that psychosocial factors can be divided into three relevant dimensions: (1) individual psychological well-being, (2) social structures, and (3) resources. Individual psychological well-being factors include psychological conditions such as mood [11,18], attitude [11,19], coping mechanism [11,17], depression [15,16,20], perceived control [13,19], and psychological distress [16,17,21]. Social structures represent the conditions of the environment in which the individual lives, including support structures [11,14,16,17], social relationships [14,18], social norms [19], and family life [22]. Finally, resources represent the means available to the individual, such as financial means, accessibility to health care [13,14], and the health service system [19].

Prior research has shown that these factors—depressive symptoms [23], poor social support, and financial stress—contribute to hospital readmission for specific patient subgroups such as those with chronic obstructive lung disease, chronic kidney disease, and heart failure [24-26]. In general, psychosocial factors could play a significant role in the hospital readmission of older adults and account for a significant proportion of the readmission risk. At the same time, psychosocial factors are indicators of a patient’s complex needs that are amenable to tailored care interventions. Such interventions can improve the patient’s clinical outcomes and reduce the utilization of health care resources.

### Literature Review

There are two conceptual models in the extant literature that link psychosocial factors to hospital readmissions for older adults. The first is Andersen’s [27] Behavioral Model of Health Services Use that posits an individual’s use of health services as a function of predisposing, enabling, and need factors. Psychosocial factors (ie, individual-level and structural-level variables) can be categorized as the model’s predisposing and enabling factors, respectively. The other is Adler and Stewart’s [28] Pathways Linking Socioeconomic Status and Health model, which suggests that environmental resources and constraints, as well as psychological influences are mechanisms that lead to health outcomes such as hospital readmission. Individual-level and structural-level psychosocial factors map to the model’s psychological and environmental variables.

In contrast to the numerous clinically related risk factors that are stored as structured data in electronic medical records (EMRs), most psychosocial factors are recorded as free text in the patient’s clinical notes such as the initial and progress clinical notes of physicians, allied health professionals, case managers, and social workers. Such unstructured textual data in the EMR represent a potentially rich and untapped source of data related to patients’ psychosocial factors. The manual extraction of psychosocial keywords from unstructured data is challenging and impractical given the copious and ever-increasing amount of clinical notes recorded in a typical EMR system. As such, there have been systematic efforts by clinicians to capture social and behavioral data, including psychosocial information, as structured data in EMR systems [29]. However, the effectiveness of these efforts in different health care contexts remains unclear. At the same time, other researchers have begun to apply text-mining techniques to efficiently extract and analyze unstructured text data in EMR clinical notes to identify these psychosocial factors.

Text-mining techniques represent a broad range of approaches for analyzing and processing semistructured and unstructured text data to construct structured data. By using powerful algorithms applied to large textual documents such as those typically found in EMR systems, text mining can “turn text into numbers” to be used for further analysis. Topic modeling, which is a specific domain in text mining that examines individual words to identify common topics and concepts, holds significant promise for extracting psychosocial factors from EMR clinical notes.

To date, only a few text-mining studies have set out to identify individuals with psychosocial factors using EMR data [30]. As such, we have limited evidence on the effectiveness of extracting psychosocial information from EMRs for the purpose of secondary health care research or routine clinical care.

### Objective

This study proposes a text-mining approach to identify older adults with key psychosocial factors obtained from clinical notes to help predict adverse health and health care utilization outcomes. To validate the efficacy of including psychological factors in the predictive model, we append these psychosocial factors to the commonly used LACE (Length of stay, Acuity of the admission, Comorbidity of the patient, and Emergency department use) Index for Readmission [31] to improve readmission risk prediction accuracy on an independent, hold-out sample of patients.

## Methods

### Design

The study was a retrospective analysis of EMR data captured by the EPIC system over a 26-month period from January 1, 2017 to February 28, 2019. Ethical approval was provided by the Domain-Specific Review Board of the National Healthcare Group, Singapore (2018/01072).

### Settings and Data Context

The sample consists of 9393 patients with 43,216 admission encounters in a 26-month period from all wards in Ng Teng Fong General Hospital, Singapore. Each clinical record was classified by the role of the author. In this sample, clinical records were authored by physicians, medical social workers, or case managers. Specifically, medical social workers and case managers are assigned to some patients who may require additional social support upon hospital admission. The dataset consists of two cohorts of patients. The first cohort includes 892 patients (3282 admission encounters) identified by the hospital as frequently readmitted patients (“Frequent Admitters” cohort). This cohort consists of patients who (1) are frequently admitted to the hospital despite having their acute medical needs met, (2) have medical conditions that require multidisciplinary care, (3) show signs of caregiver stress, (4) encounter frequent falls (more than two falls in the last 12 months) and require functional management at home, or (5) face medication management issues (eg, noncompliance to their medication regime). The second cohort consists of 9377 randomly selected patients (39,934 admission encounters) admitted to the hospital (“Standard” cohort). The “Standard” cohort consists of patients admitted to the hospital’s inpatient wards during the sampling period. The purpose of including the “frequent admitters” cohort was to oversample the frequent readmission cases and facilitate the training of the text-mining algorithm to extract psychosocial topics often associated with readmission risks. This type of oversampling method is commonly applied in health care research to train machine-learning algorithms [32,33]. The combined cohorts were randomly split into a training dataset and a hold-out/test dataset to ensure that both the training and test dataset had similar distributions of patients in the “frequent admitters” and “standard” cohorts. The training dataset comprised 30,252 admission encounters and the hold-out/test dataset comprised 12,964 admission encounters. The unit of analysis is each admission encounter.

**Figure 1:**
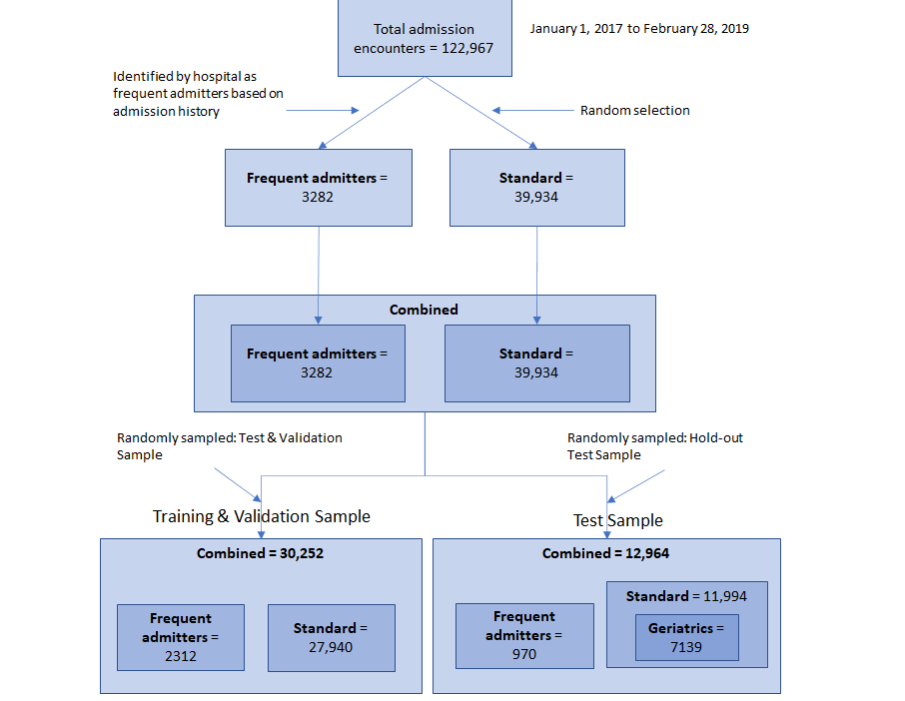
Sampling methodology. All values represent the admission encounters. For the “frequent admitters” cohort (3282 encounters), there are 892 unique patients and for the “standard” cohort (39,934 encounters), there are 9377 unique patients. The “geriatrics” cohort represents a subsample of patient encounters within the “standard” cohort where the age of the patient at time of encounter is greater or equal to 65 years.

We used the 10-fold cross-validation method to train and validate the model with the training dataset. The validated model was subsequently tested against the hold-out/test dataset in four ways. First, we used the “Combined” test dataset, which is the dataset compiled to have a similar distribution as the training dataset, containing proportionally more “frequent admitters” to ensure that the model was tested using a similar distribution of patients as that used to train the model. Second, we used the “Standard” test dataset, which was the sample randomly drawn among patients from the hospital; this sample represents a typical patient that a hospital encounters. We used this dataset to test for the generalizability of the model and to rule out overfitting. Third, we used a “Frequent” test dataset, consisting of “frequent admitters,” which was mainly used to test the fit of the model in predicting frequent admitters, as a key concern for many hospitals. Finally, we used a “Geriatrics” test dataset, comprising only geriatric patients (≥65 years old selected from the “standard” cohort test dataset), to test if the model works for the geriatric specialty where it is likely to be deployed. Additional details and procedures of cohort selection are shown in Figure 1.

### Data Processing and Algorithm Development

For *each* admission encounter, we combined the clinical notes written by authors with similar roles (eg, all notes written by physicians were combined as physician’s notes). The notes were combined based on the author’s role (physician, medical social worker, and case manager) because each role would potentially document similar issues. Hence, it is more efficient to mine the unstructured clinical notes for each role to identify common or similar topics. We combined the notes for each admission encounter instead of analyzing each note entry as the unit of analysis because a patient’s psychosocial conditions are less likely to vary for each admission encounter.

We then applied natural language processing text mining to the clinical notes in the training dataset. We used the latent Dirichlet allocation (LDA) topic modeling algorithm to extract the common topics present in the clinical notes and then numerically weighed each topic’s intensity (loadings) in the clinical notes. A vector of lexicographically related words represents each *topic* due to the frequent occurrence of these words in proximity across different notes. A high *loading* value represents the presence of the topic in the clinical note. This process was performed separately for the physician, medical social worker, and case manager notes. A total of 100 topics were extracted from each set of notes based on the clinician’s role (ie, physician, medical social worker, case manager).

Two geriatric specialists reviewed and classified these 100 topics into broader themes, specifically dividing them into psychosocial issues or nonpsychosocial-related issues. Additionally, we conducted four interviews with a group of medical social workers and case managers to triangulate if this classification is appropriate. It is important to note that this added classification into broader themes by clinicians is solely to facilitate the reporting and interpretation of results. These broader themes were *not used* in the subsequent development of the readmission risk model, and only the LDA classification loadings were used in the training of the readmission risk model. Further details of the text mining procedure are provided in Multimedia Appendix 1.

We combined the topic’s intensity (loadings) for each set of notes with structured predictors of readmission established in the LACE Index for Readmission as predictors for estimating readmission risk. As readmission risk is a function of various factors beyond psychosocial factors, we incorporated the LACE index to take into account some of the factors reported in the literature. The LACE index is a score commonly used to predict a patient’s 30-day hospital readmission risk [31]. The index consists of the following variables: (1) the length of stay (L), (2) the acuity of the current or previous admission (A), (3) comorbidities of the patient as measured by the Charlson Comorbidity Index score (C), and (4) the number of visits to the emergency department in the preceding 6 months (E).

The readmission risk model was fitted using the gradient boosting trees (GBT) algorithm to predict the outcome of readmission within the next 30 days from the discharge date of the current admission. GBT uses an ensemble of multiple trees to generate more accurate prediction models for classification and regression. The algorithm’s premise is to build a series of trees, where each tree is trained with the objective to correct the misclassification errors of the previous tree in the series.

We tested the model’s predictive accuracy using the four different hold-out test samples described above. To assess the predictive value of the clinical notes, we fitted a LACE baseline readmission model *without* using the topics from the notes. We then compared this baseline model against models that include the physician notes and social notes (ie, medical social worker notes and case manager notes) jointly and separately.

## Results

### Evaluating the Predictive Value of Psychosocial Information

As expected, we observed that physicians record fewer psychosocial issues than medical social workers and case managers (Table 1). The more detailed distribution of the specific topics extracted is provided in Tables A1-A3 of Multimedia Appendix 1.

The descriptive statistics of the variables used in the readmission risk model for each test cohort are provided in Table 2.

**Table 1:** Distribution of psychosocial topics (N=100).

Role of author	Proportion of psychosocial topics, n (%)	Proportion of nonpsychosocial topics, n (%)
Physician	25 (25)	75 (75)
Medical social worker	100 (100)	0 (0)
Case manager	88 (88)	12 (12)

**Table 2:** Descriptive statistics of variables in the LACE (Length of stay, Acuity of the admission, Comorbidity of the patient, and Emergency department use) readmission model (patient encounter level).

Variable	Frequent cohort^a^, mean (SD)	Standard cohort^b^, mean (SD)	Geriatrics cohort^c^, mean (SD)	Combined cohort^d^, mean (SD)
Age (years)	72.94 (13.24)	67.07 (15.98)	77.62 (8.09)	67.51 (15.87)
Gender (1: Male, 0: Female)	0.50 (0.50)	0.55 (0.50)	0.50 (0.50)	0.54 (0.50)
Length of stay (days)	6.73 (13.18)	5.47 (10.15)	6.65 (10.81)	5.57 (10.41)
Charlson Comorbidity Index	0.47 (1.39)	0.41 (1.18)	0.49 (1.38)	0.42 (1.20)
Emergency department admission (1: Yes, 0: No)	0.50 (0.50)	0.57 (0.50)	0.58 (0.49)	0.56 (0.50)
Intensive care unit stay (1: Yes, 0: No)	0.03 (0.17)	0.05 (0.22)	0.04 (0.21)	0.05 (0.22)
Emergency department visits in last 6 months	2.86 (3.07)	1.39 (2.77)	1.47 (2.44)	1.50 (2.82)

^a^Patients identified by the hospital as frequent readmission patients.

^b^Sample of a typical hospital patient.

^c^Subset of patients in the “Standard” sample who are 65 years of age or older.

^d^Combination of the “Frequent” and “Standard” samples.

The area under the receiver operating characteristic curve (AUROC) of the LACE baseline predictive model ranged from 0.8288 to 0.8397 (Table 3) for the four different test cohorts (Frequent, Standard, Geriatrics, and Combined). The baseline model only considered common factors identified in the prior literature associated with readmission risks and did not include psychosocial factors extracted from the clinical notes. The receiver operating characteristic curve is a plot representing the diagnostic ability of a binary classifier while varying the discriminatory threshold (ie, the cut-off value to reclassify one state to the other). With varying discriminatory threshold values, the different sets of true positive rate (sensitivity) are plotted against the corresponding false positive rates (1–specificity). Thus, AUROC is a representation of the overall performance of the classifier.

Adding the text-mined notes from the medical social workers and case managers increased the AUROC of the model to 0.8573-0.8707. Further appending the clinical notes from physicians increased the AUROC to 0.8952-0.9100.

**Table 3:** Results of the readmissions prediction model.

Model		AUROC^a^	Sensitivity	Specificity	PPV^b^	NPV^c^
**LACE^d^ baseline**						
	Frequent^e^	0.8288	0.7021	0.7840	0.7466	0.7438
	Standard^f^	0.8302	0.7341	0.7606	0.6649	0.8156
	Geriatrics^g^	0.8254	0.7479	0.7328	0.6713	0.7994
	Combined^h^	0.8397	0.7303	0.7757	0.6696	0.8221
**LACE baseline+social^i^**						
	Frequent	0.8573	0.7598	0.7573	0.7394	0.7767
	Standard	0.8621	0.7661	0.7796	0.6922	0.8375
	Geriatrics	0.8686	0.7749	0.7832	0.7228	0.8267
	Combined	0.8707	0.7763	0.7825	0.6896	0.8490
**LACE baseline+physician^j^+social**						
	Frequent	0.8952	0.8232	0.8136	0.8001	0.8354
	Standard	0.9001	0.8224	0.8235	0.7509	0.8776
	Geriatrics	0.9100	0.8318	0.8331	0.7843	0.8716
	Combined	0.9069	0.8254	0.8318	0.7534	0.8845

^a^AUROC: area under the receiving operating characteristic curve.

^b^PPV: positive predictive value.

^c^NPV: negative predictive value.

^d^LACE: Length of stay, Acuity of the admission, Comorbidity of the patient, and Emergency department use.

**^e^**Hold-out sample of patients identified by the hospital as frequent readmission patients.

^f^Hold-out sample of a typical hospital patient.

^g^Subset of patients in the “Standard” sample who are 65 years or older.

^h^Combination of “Frequent” and “Standard” hold-out samples.

^i^Social represents the text-mined notes that medical social workers and case managers provided.

^j^Physician represents the text-mined notes provided by physicians.

### Comparison Across Patient Profiles

The addition of textual information improved the AUROC of the readmission model. This improvement was particularly more significant for geriatric patients than for other cohorts of patients (Table 4). For geriatric patients, notes from the medical social workers and case managers improved the AUROC by 4.32%. Combining these notes with physician notes further improved the AUROC by 8.46% compared with the baseline LACE readmission model.

**Table 4:** Improvements of prediction (area under receiver operating characteristic curve) over the baseline LACE (Length of stay, Acuity of the admission, Comorbidity of the patient, and Emergency department use) model for different test cohorts.

Notes	Frequent cohort^a^	Standard cohort^b^	Geriatrics cohort^c^	Combined cohort^d^
Social^e^	2.85%	3.19%	4.32%	3.10%
Social and physician^f^	6.64%	6.99%	8.46%	6.72%

^a^Hold-out sample of patients identified by the hospital as frequent readmission patients.

^b^Hold-out sample of a typical hospital patient.

^c^Subset of patients in the “Standard” sample who are 65 years or older.

^d^Combination of “Frequent” and “Standard” hold-out samples.

^e^The readmission model with clinical notes from the medical social worker and case manager.

^f^The readmission model with clinical notes from the medical social workers and case managers.

## Discussion

### Principal Findings

The AUROC of our readmission risk model was higher than the typical accuracy of readmission predictive models, ranging from 0.66 to 0.83, as reported in an earlier review of 30 studies [6]. The results also suggest that the readmission predictive algorithm’s performance for all four cohorts (frequent admitters, standard, geriatrics, and the combination of frequent and standard groups) are relatively similar. Thus, this model can be applied to geriatric patients as the typical pool of patients who require additional management for readmission risks. Further, when taking into account the psychosocial information captured by nonphysicians (ie, medical social workers and case managers) by adding social topics, the prediction accuracy improved by 0.0285-0.0432. When we added the physicians’ textual clinical notes, the AUROC further increased by 0.0362-0.0414 in different cohorts.

Overall, the results show that with the addition of text-mined clinical notes from physicians and other clinicians, the AUROC of readmission prediction improves by 0.0664 to 0.0842, suggesting the added benefits of extracting psychosocial information from textual clinical notes in predicting readmission risk.

This study shows that clinicians could leverage natural language processing to gain more information from the EMR system beyond the traditional structured data commonly used to predict readmission risk. Specifically, this study establishes a proof of concept for the use of text-mining techniques with EMR unstructured free text to identify psychosocial predictors of hospital readmission, particularly among geriatric patients. In doing so, our findings support the viability of the psychosocial approach in potentially reducing readmission rates. Thus, our study represents a T2 translational stage (to patients) of research, paving the way toward the T3 translational stage (to practice). In terms of development along the translational pathway, the next phase will focus on proof of value of embedding text-mining techniques in prediction models used to identify the risk of early readmission among hospitalized patients. The purpose of this phase is to perform a comprehensive geriatric assessment for high-risk patients with the goal of offering tailored care management. By managing patients’ specific physical and psychosocial needs, we should observe improvement in the quality of care and a reduction in unnecessary health care utilization. In this way, precious heath care resources can be optimally allocated to patients who will obtain the greatest benefit. This strategy is particularly relevant for older hospitalized patients, who are more likely to have unmet psychosocial needs and for whom our augmented risk prediction model performs the best. To achieve proof of value, future research could use quasiexperimental designs to compare the feasibility and effectiveness of a product that combines text-mined psychosocial factors in a state-of-the-art prediction model with those of a product that only has a prediction model.

Beyond the application of text-mining techniques to the prediction of hospital readmission, this study also presents the broader and extended possibility of using the same technical approach developed for the EMR to identify a set of underdiagnosed clinical conditions in older adults, which will have an important influence on their health and health care utilization outcomes.

### Conclusion

Psychosocial profiles of patients can be curated and quantified from text mining clinical notes, and these profiles can be successfully applied to artificial intelligence models to predict readmission risks. The use of text mining improved the accuracy of predicting readmission, and this improved predictive accuracy was higher for geriatric patients than for other patient cohorts.

## Appendix

Multimedia Appendix 1 Detailed methods of text mining approach and Tables A1-A3.

## Abbreviations

AUROC: area under the receiver operating characteristic curve
EMR: electronic medical record
GBT: gradient boosting trees
LACE: Length of stay, Acuity of the admission, Comorbidity of the patient, and Emergency department use
LDA: latent Dirichlet allocation
