# Supplementary material for: Prediction of Readmission in Geriatric Patients From Clinical Notes: Retrospective Text Mining Study
Source: J Med Internet Res. 2021 Oct 19;23(10):e26486. doi: 10.2196/26486 (PMC8564665; doi:10.2196/26486)
Supplement: Multimedia Appendix 1 [file jmir_v23i10e26486_app1.pdf]

**Textmining Procedure**

The purpose of the natural language processing (NLP) analysis is to develop an efficient and robust way to extract, analyze, and systematically interpret the notes. Using machine learning (details below), free-form progress notes are translated into various themes that will be empirically validated to be associated with readmission. The steps taken to anonymize and prepare the progress notes for NLP analysis are as follows:

1) Progress notes of patients identified in the abovementioned cohort will be downloaded from the EMR database.
2) A parsing script is applied to all the progress note to ensure that any free-form text which matches the patient's name will be removed. Further, a filtering script will remove any name following standard prefixes (Mr., Mrs., Ms., Mdm., Dr.). The resulting dataset will consist of progress notes that do not contain any information to identify the patient to be in line with the Health Insurance Portability and Accountability Act (HIPAA) and Human Biomedical Research Act (Singapore) to maintain patient's confidentiality.
3) The final working database (WD) has this schematic structure:
    a. patient ID tag (de-identified) + de-identified clinical patient progress notes + relevant clinical measurements (e.g. LACE readmission risk indicators).
4) The final working database is randomly split into a training/ validation dataset and a hold-out, test dataset.
5) **Training and Extracting the Text Topics:** In the training/ validation dataset an NLP algorithm will be applied to the de-identified patient progress notes. Training of the NLP algorithm consists of the following sub-steps:
    a. Text parsing: all words in the progress notes will first be lemmatized into their root form (e.g. "happiness" becomes "happy").

b. Part-of-speech (POS) tagging: all lemmatized words will be tagged based on their role in the sentence (e.g. noun, adverb, adjective etc.)
c. Establishing of stop words, filtering and weighting: words which have the least information quality such as conjunction, will be dropped and the rest of the words will be weighted and filtered based on their information quality to facilitate the text mining. Filtering the terms also ensures that terms that appear excessively can be weighted downwards – this is to minimize the effect of patients with a poorer prognosis, causing physicians to be added cautious and document more words in the clinical notes.
d. Latent Dirichlet Allocation algorithm applied to classify thez words into topics and compute a loading weight on each topic representing the intensity of the topic in the clinical note. This “inductive” approach presents the common topics that exist in the dataset. Subsequently, a lexicon is built using these topics, represented by a vector of thematically similar words.
e. A “deductive” text mining approach will be applied to score each patient in the hold-out, test dataset described in the next point.

6) **Testing the Text Topics:** We measure the similarity of the notes in the test dataset to the lexicon of topics established in the training dataset. Each note in the test dataset is subjected to the same text parsing, POS tagging, stop words filtering, and weighting described earlier. We transformed the words in each note into a vector and compared it against the training model’s lexicon, and the intensity of each topic is measured. The measured intensity for each topic in the test topic is used as predictors for the readmission risk model.
7) **Classification of Themes:** For further interpretation, two physicians further classify the 100 topics into broader themes. Note that the readmission algorithm does not require this

added classification step, and this classification is solely for the purpose of having an additional interpretation of the topics. The broader themes are listed in Tables A1 to A3 below.

**Table A1: Themes Extracted from Physician Clinical Notes**

| Themes | Topic Count | Psychosocial |
|---|---|---|
| Medical Condition | 41 | No |
| Clinical note | 8 | No |
| Physical exam | 7 | No |
| Patient Location (residence) | 6 | Yes |
| Medication instructions (discharge) | 6 | Yes |
| Blood investigations | 5 | No |
| Caregiver | 4 | Yes |
| Medical Speciality | 4 | No |
| Body measures | 3 | No |
| Discharge plan | 3 | Yes |
| Medication | 3 | No |
| Others | 3 | Yes |
| Physical Function | 2 | No |
| Admission instructions | 2 | No |
| End of Life instructions | 1 | Yes |
| Financial | 1 | Yes |
| Travel history | 1 | Yes |

**Table A2: Themes Extracted from Medical Social Worker Clinical Notes**

| Themes | Topic Count | Psychosocial |
|---|---|---|
| Discharge plan | 22 | Yes |
| Family | 18 | Yes |
| Caregiver | 15 | Yes |
| Financial | 12 | Yes |
| Others | 7 | Yes |
| Equipment | 5 | Yes |
| Advanced care planning | 4 | Yes |
| Agency & welfare services | 4 | Yes |
| Communication | 4 | Yes |
| Meals | 3 | Yes |
| Mental | 3 | Yes |
| Renal home care | 2 | Yes |

| | | |
|---|---|---|
| Physical Function | 1 | Yes |

**Table A3: Themes Extracted from Case Manager Clinical Notes**

| Themes | Topic Count | Psychosocial |
|---|---|---|
| Medication (instructions) | 52 | Yes |
| Instructions (medical) | 9 | Yes |
| Pharmacist | 9 | Yes |
| Med. Condition | 8 | No |
| Others | 6 | Yes |
| Case Management | 3 | Yes |
| Instruction | 3 | Yes |
| Intructions (procedure) | 3 | Yes |
| Renal | 3 | No |
| Caregiver | 2 | Yes |
| Financial | 1 | Yes |
| Smoking cessation | 1 | No |
